# Impact of shortened length of stay for delivery on the required bed capacity in maternity services: results from forecast analysis on administrative data

**DOI:** 10.1186/s12913-019-4500-8

**Published:** 2019-09-05

**Authors:** Mélanie Lefèvre, Koen Van den Heede, Cécile Camberlin, Nicolas Bouckaert, Claire Beguin, Carl Devos, Carine Van de Voorde

**Affiliations:** Belgian Health Care Knowledge Centre (KCE), Doorbuilding, Boulevard du Jardin Botanique 55, 1000 Bruxelles, Belgium

**Keywords:** Hospital capacity, Length of stay, Maternity beds

## Abstract

**Background:**

We examine the implications of reducing the average length of stay (ALOS) for a delivery on the required capacity in terms of service volume and maternity beds in Belgium, using administrative data covering all inpatient stays in Belgian general hospitals over the period 2003–2014.

**Methods:**

A projection model generates forecasts of all inpatient and day-care services with a time horizon of 2025. It adjusts the observed hospital use in 2014 to the combined effect of three evolutions: the change in population size and composition, the time trend evolution of ALOS, and the time trend evolution of the admission rates. In addition, we develop an alternative scenario to evaluate the impact of an accelerated reduction of ALOS.

**Results:**

Between 2014 and 2025, we expect the number of deliveries to increase by 4.41%, and the number of stays in maternity services by 3.38%. At the same time, a reduction in ALOS is projected for all types of deliveries. The required capacity for maternity beds will decrease by 17%. In case of an accelerated reduction of the ALOS to reach international standards, this required capacity for maternity beds will decrease by more than 30%.

**Conclusions:**

Despite an expected increase in the number of deliveries, future hospital capacity in terms of maternity beds can be considerably reduced in Belgium, due to the continuing reduction of ALOS.

**Electronic supplementary material:**

The online version of this article (10.1186/s12913-019-4500-8) contains supplementary material, which is available to authorized users.

## Background

Reductions in the length of postpartum hospital stay are observed in almost all industrial countries. In the United States, for instance, the standard length of postpartum stay was 8 to 14 days in the 1950s but declined to less than 2 days in the mid-1990s and levelled off at 2 days ever since [1, 2]. Also in Europe the average length of stay (ALOS) for a normal delivery dropped from 5 days in 2000 to 3.2 days in 2014 [3]. Belgium, the country that is analysed in the current study, follows this international evolution with a reduction in the average length of stay for a normal delivery of almost 25% since 2000 (from 4.9 days in 2000 to 3.7 days in 2014) [1].

Aside from evolutions in clinical practice, reducing the average length of stay is perceived as a way to achieve efficiency [3, 4]. Indeed, a shorter stay reduces the cost per patient as it shifts care from inpatient to less expensive post-acute settings. While controversy remains [5], early postpartum discharge appears to be safe in controlled studies with a post-hospital follow-up program [6]. Typically, reducing the length of time patients stay in hospital could release capacity in the hospital system.

Due to this reduction in length of stay, several countries, such as France [7] and England [8], have reduced their bed capacity in maternity services. However, this process is often the result of negotiation and is based on very rough data. To avoid under- and overcapacity, it is necessary to provide policy makers with a more objective and detailed assessment of the required capacity.

Belgium is a relevant case for at least two reasons. First, the country is currently undergoing a major reform of the hospital landscape and payment system in which maternity services are an important component [9]. In the context of this reform, several pilot projects focusing on deliveries with shortened stays have been launched [10]. There is indeed room for such a shortening as the ALOS is situated 20% above the European average and well above the 2 days threshold after which further reductions appear hard to achieve [1]. Second, almost all deliveries (99%) take place in the hospital [11].

We examine the possible implications of reducing the ALOS on the required capacity in terms of service volume and maternity beds in Belgium. We first shortly describe the currently available capacity of maternity services. Then, we generate forecasts for the required capacity with a time horizon of 2025.

## Methods

Although the focus of this study is on maternity services, the forecast analysis is part of a larger exercise exploring the global required hospital bed capacity [12]. In order to support decision makers in anticipating future hospital capacity need (in terms of stays, days and beds), a projection model was built that generates forecasts of all inpatient and day-care services in general hospitals in Belgium with a time horizon of 2025. However, the future required capacity for maternity services was examined more into detail and is the focus of this article.

The starting point of the projection model is hospital use in 2014. This is subsequently adjusted by the combined effect of three evolutions to generate forecasts for future hospital capacity: the future evolution in population size and composition, the time trend evolution of ALOS, and the time trend evolution of the admission rates (more details below). The effect of the three evolutions can be separately identified.

The outcome of the projection model can be interpreted as a ‘no policy change scenario’. Nevertheless, policy actions that were taken in the past, are reflected in the estimated time trends and their continued effect is projected into the future. In addition, we develop an alternative scenario in which the impact of an accelerated reduction of ALOS for deliveries is evaluated.

### Data

#### Population data

Past demographics are necessary to analyse hospital service use by sociodemographic group over time. The observed population size by age and sex over the period 2003 to 2016 is provided by Statistics Belgium based on the place of residence and aggregated at the level of the three Belgian regions: Flanders, Wallonia and Brussels.

For the future evolution of the population residing in Belgium we use the projections that were released by the Federal Planning Bureau and Statistics Belgium in March 2017, providing annual projections of the population size up to 2061, subdivided by age, sex and region [13]. These population projections take into account international migration, domestic relocation, and the future evolution in fertility and mortality.

#### Hospital data and APR-DRG classification system

All Belgian general hospitals are required to submit twice a year a large set of data on all inpatient and day-care stays and emergency room contacts. Hospital data were available for 2003–2014 for all inpatient stays.

Each inpatient stay is assigned an APR-DRG (All Patient Refined-Diagnosis Related Group) code using information on principal diagnosis, secondary diagnoses and procedures, birthweight (for newborns), age of the patient, etc.. The basic structure is extended by adding severity of illness (SOI) subclasses to each APR-DRG. Severity of illness is defined as the extent of physiologic decompensation or organ system loss of function and introduces 4 categories for SOI: 1 (minor), 2 (moderate), 3 (major) and 4 (extreme).

### Model

#### Projection model

The projection model generates hospital capacity forecasts expressed in number of stays, number of nursing days and number of beds with a time horizon of 2025. It adjusts the observed hospital use in 2014 to the combined effect of three evolutions: the change in population size and composition, the time trend evolution of ALOS, and, the time trend evolution of the admission rates. A detailed description of the model can be found in Van de Voorde et al. (2017) [12] and in Additional file 2.

First, the projections for hospital use were adjusted for the evolution in population size of a sociodemographic group, defined by sex, seven age groups and three regions.

Second, using data over the period 2003 to 2014, time trends were computed up to 2025 for the length of stay by APR-DRG-SOI, and the admission rates by age group and APR-DRG-SOI. Length of stay is defined as the number of days between admission and discharge. Admission rates are expressed in stays per 100,000 individuals in the relevant subgroup. A wide range of potential specifications was used and a two-step evaluation procedure that not only assesses the model’s ability to fit historic patterns but also its potential to produce accurate outcomes.

In a first step of the trend estimation, the hospital data were transformed in a quarterly series of ALOS and incidence rates for each subgroup. A time trend was estimated using deterministic – exponential, logarithmic, linear and power – and ARIMA (auto-regressive integrated moving average) models. The latter are a general class of stochastic time series models – the random walk model and exponential smoothing models are well-known special cases – without pre-determined functional form [14–16]. Next, each specification was evaluated using the Akaike Information Criterion for small samples (AICc) to assess goodness of fit to historical data and the level of complexity [17, 18]. We preserved the best model as well as competing models whose AICc value is only marginally different (difference less than 5 units) [19].

In a second step, selected models were re-estimated on a subpart of the data (2003–2011) and predictions were made for an evaluation period (2012–2014). The prediction’s accuracy was assessed using the Mean Absolute Error (MAE). We calculated the mean and standard deviation of the MAE over all remaining models. The final selection consists of models with a MAE below the mean value augmented with one standard deviation.

The final projection outcome was calculated as the average of the remaining estimates. The use of an average forecast is a popular strategy in time series analysis to improve accuracy and reduce the influence of occasional extreme projections [16, 20]. Moreover, through the use of ARIMA models and a validation period, our methodology gives more weight to recent observations.

The estimated time trends identify long-run time patterns in currently available (historical) data that are assumed to continue into the future. This should be interpreted broadly since the estimated time trends are able to jointly capture epidemiological trends, medico-technical progress, development of community care, the ongoing development in medical practice and organization, the influence of financial incentives and other policy decisions, etc. Our projections show e.g. an important shortening in ALOS for both vaginal delivery and caesarean delivery (C-section), but at a faster pace for SOI-levels 1 and 2 than for levels 3 and 4. The trend in admission rates uncovers three important patterns: a shift from vaginal delivery towards C-section; a shift in APR-DRG 560 (vaginal delivery without procedure) from SOI 1 to SOI 2 at all ages; and a much more important increase in birth rates for women aged above 35 years compared to women aged below 35 years.

#### Capacity indicators: stays, days and beds

The volume of hospital stays accounts for the change in population size and composition and admission rates. More specifically, the future number of stays for a certain pathology group (e.g. vaginal delivery) and demographic group (e.g. women aged 35–45) in a specific year (e.g. 2023) corresponds to the number of hospital stays observed in 2014 multiplied by the relative change in population size and admission rates for that group of women between 2014 and 2023.

The projected number of nursing days for that specific year is computed by multiplying the projected number of stays for that year by the ALOS observed in 2014 adjusted by the change rate since 2014.

The number of nursing days represents an enlightening metric for capacity planning, as some countries, such as England and France, are moving towards planning with respect to service volume and activity. Nevertheless, despite several limitations, bed number is still the most used metric for capacity planning [21].

To calculate the future number of beds we compute the projected number of nursing days and apply a specific occupancy rate to infer the future bed need (Number of beds = Number of inpatient nursing days / (365 x occupancy rate)). Occupancy rates generally range from 70 to 90%. The rate we used for maternity beds is 70% which is also the rate applied in the Belgian hospital payment system [22]. Although there is no consensus in the literature and different sizes and types of beds have different optimum average occupancy, hospitals generally aim for a rate of 80 to 85% [23, 24]. To manage peak demand, occupancy rates are often lower.

### Alternative scenario

Given the political willingness to reform maternity services in Belgium, a ‘no policy change scenario’ forecast might not reflect the future. Moreover, we observe that the length of stay for a delivery in Belgium is long compared to some other countries [3]. Therefore, as an alternative to using statistical forecasts for ALOS, we evaluate the potential effects of an accelerated reduction in ALOS for vaginal deliveries (excluding sterilization, dilatation, curettage or complicating procedure, APR-DRG 560) and C-sections (APR-DRG 540) for SOI 1 and 2, applying international benchmarks in ALOS as future targets.

The chosen international benchmarks are based on good performing OECD countries (e.g. Sweden) and current levels observed in the US. For the latter, we used two data sources with recent figures and an APR-DRG-based classification system as in Belgium, which can easily be compared to the Belgian APR-DRG classification for childbirth: the observed ALOS in 2016 in the US state Texas and the ALOS from the Healthcare Cost and Utilization Project (HCUP) of the US Agency for Healthcare Research and Quality (AHRQ) [25, 26]. The following benchmarks are applied:

- APR-DRG 560, SOI 1: an ALOS of 2 days in 2025
- APR-DRG 560, SOI 2: an ALOS of 2.5 days in 2025
- APR-DRG 540, SOI 1: an ALOS of 3 days in 2025
- APR-DRG 540, SOI 2: an ALOS of 4 days in 2025

The international benchmarks are gradually introduced in the model: until 2017 the projected evolution in ALOS is kept and between 2018 and 2025 a linear evolution towards the benchmark value is assumed.

## Results

### Current situation

#### A high density of mainly small-sized maternity services

Amongst the 103 general hospitals in Belgium, nearly all (98) have at least a maternity service, some have more than one, spread on several sites: 3176 licensed maternity beds are divided among 111 different hospital sites (2014). The size of maternity services differs across regions; this can be illustrated by the proportion of small-sized maternity services (with 15 or less licensed beds): Flanders (23.4%) and Wallonia (19.4%) have a higher proportion than Brussels where it is only 9% (1 out of 11 maternity services).

#### National overcapacity with regional imbalances

The number of deliveries per site is highly variable (ranging from 212 to 3333) with a median of 897 deliveries per site which roughly corresponds to 2.5 deliveries per day.

In 2014, there were 147,547 inpatient stays in a maternity bed in Belgium, corresponding to 650,302 nursing days. Based on this observed number and a bed occupancy rate of 70%, the estimated need for maternity beds is 2545 beds (650,302 / (365 × 0.7)). Compared to the 3176 licensed beds, there is an overcapacity of 20%. This overcapacity is reflected in the occupancy rates per site. Except in Brussels, maternity services have very low occupancy rates. On a national level, the average occupancy level of the available capacity of licensed maternity beds was below 50% in 2014. Fluctuations over the course of a year range between 39.9 and 58.3%. This is a clear indication that there are too many licensed maternity beds in Belgium.

### Baseline forecast

#### Number and type of deliveries

The number of inpatient stays for delivery is expected to increase from 122,563 in 2014 to 127,970 in 2025 (a 4.4% increase). Based on the past evolution in medical practice in Belgium, we forecast a shift towards more C-sections in comparison to the number of vaginal deliveries. Additional file 1 shows the forecasted evolution of the rate of C-sections in Belgium and its three regions.

#### Number of stays on maternity services

The two largest groups of maternity patients are women delivering, either vaginally or by C-section. However, 16.9% of the registered stays that (partly) take place in maternity services concern an admission that does not concern a delivery. In order to have a complete view of the required capacity in maternity services, we also have to include these stays. Doing so, the number of inpatient stays in a maternity bed in Belgium is expected to increase to 153,267 in 2025, that is, an increase of 3.9% with respect to the 2014 situation.

#### Average length of stay

**Fig. 1:**
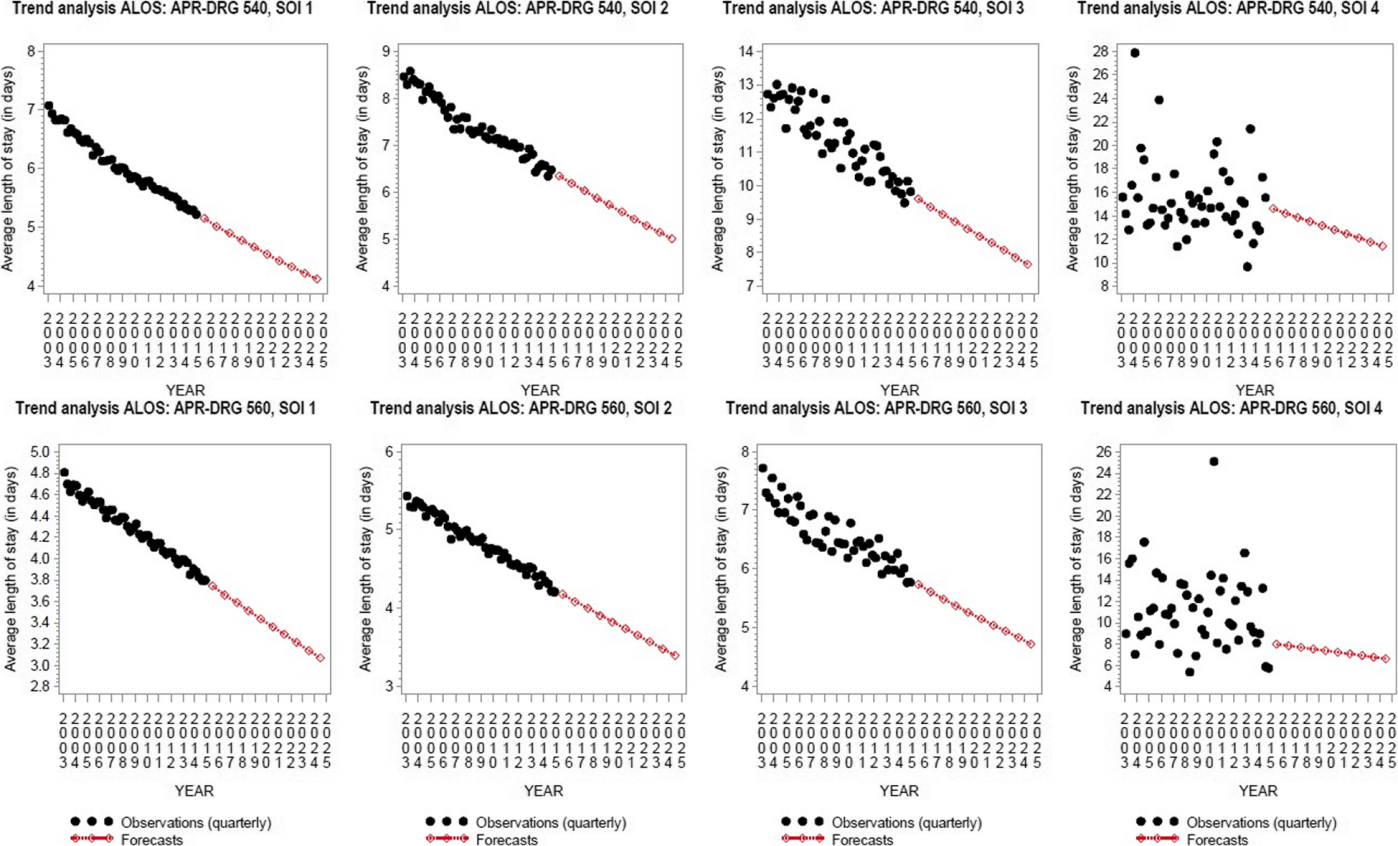
ALOS trend analysis for C-section deliveries (APR-DRG 540) and vaginal deliveries without procedure (APR-DRG 560) (2003–2025)

Figure 1 shows forecasts generated by the projection model for the ALOS for C-section (APR-DRG 540) and for vaginal delivery without procedure (APR-DRG 560). For both types of deliveries, a reduction in ALOS is projected. The ALOS decreases from 3.8 days in 2014 to 3 days in 2025 for vaginal delivery without procedure with SOI 1 and from 4.3 days to 3.3 days for SOI 2. For C-section stays, the ALOS is projected to decline from 5.3 days in 2014 to 4 days in 2025 for SOI 1 and from 6.5 days to 4.9 days for SOI 2.

#### Required capacity

Table 1 presents the baseline forecast results from the projection model. The required number of maternity beds is projected to decrease by 17.0% (432 beds) between 2014 and 2025. Compared to the number of licensed beds in 2014 (3176 beds based on information from December 2014 provided by the Federal Public Service of Health), the necessary reduction is even bigger. Indeed, as there was already a substantial overcapacity in 2014 (631 more licensed beds than required), this overcapacity will continue to grow (up to 1063 beds in 2025) if no additional policy action is undertaken.

**Table 1:** Baseline forecast results for inpatient stays and days

	2014	2020	2025	Abs. difference 2014–2025	Rel. difference 2014–2025
Total inpatient stays	1,851,612	1,958,563	2,072,756	221,144	11.9%
Total stays in maternity beds	147,547	150,871	153,267	5720	3.88%
APR-DRG 540	26,144	28,400	30,130	3986	15.2%
APR-DRG 541	346	337	323	−23	−6.6%
APR-DRG 542	272	260	249	−23	−8.5%
APR-DRG 560	95,801	96,687	97,268	1467	1.5%
Other APR-DRGs	24,984	25,187	25,297	313	1.3%
Total inpatient days	12,906,895	12,446,613	12,268,831	− 638,064	−4.9%
Total days in maternity beds	650,302	591,060	539,958	− 110,344	−17.0
APR-DRG 540	158,551	148,570	138,679	−19,872	− 12.5%
APR-DRG 541	1793	1532	1339	− 454	−25.3%
APR-DRG 542	1438	1235	1087	− 351	− 24.4%
APR-DRG 560	391,268	350,357	316,330	− 74,938	− 19.2%
Other APR-DRGs	97,252	89,366	82,523	− 14,729	−15.1
Maternity beds	2545	2313	2113	− 432	−17.0%

APR-DRG 540 ‘Caesarean Delivery’, APR-DRG 541 ‘Vaginal Delivery with Sterilization and/or Dilatation and Curettage’, APR-DRG 542 ‘Vaginal Delivery with Complicating Procedure except Sterilization and/or Dilatation and Curettage’, APR-DRG 560 ‘Vaginal Delivery’

### Alternative scenario

**Fig. 2:**
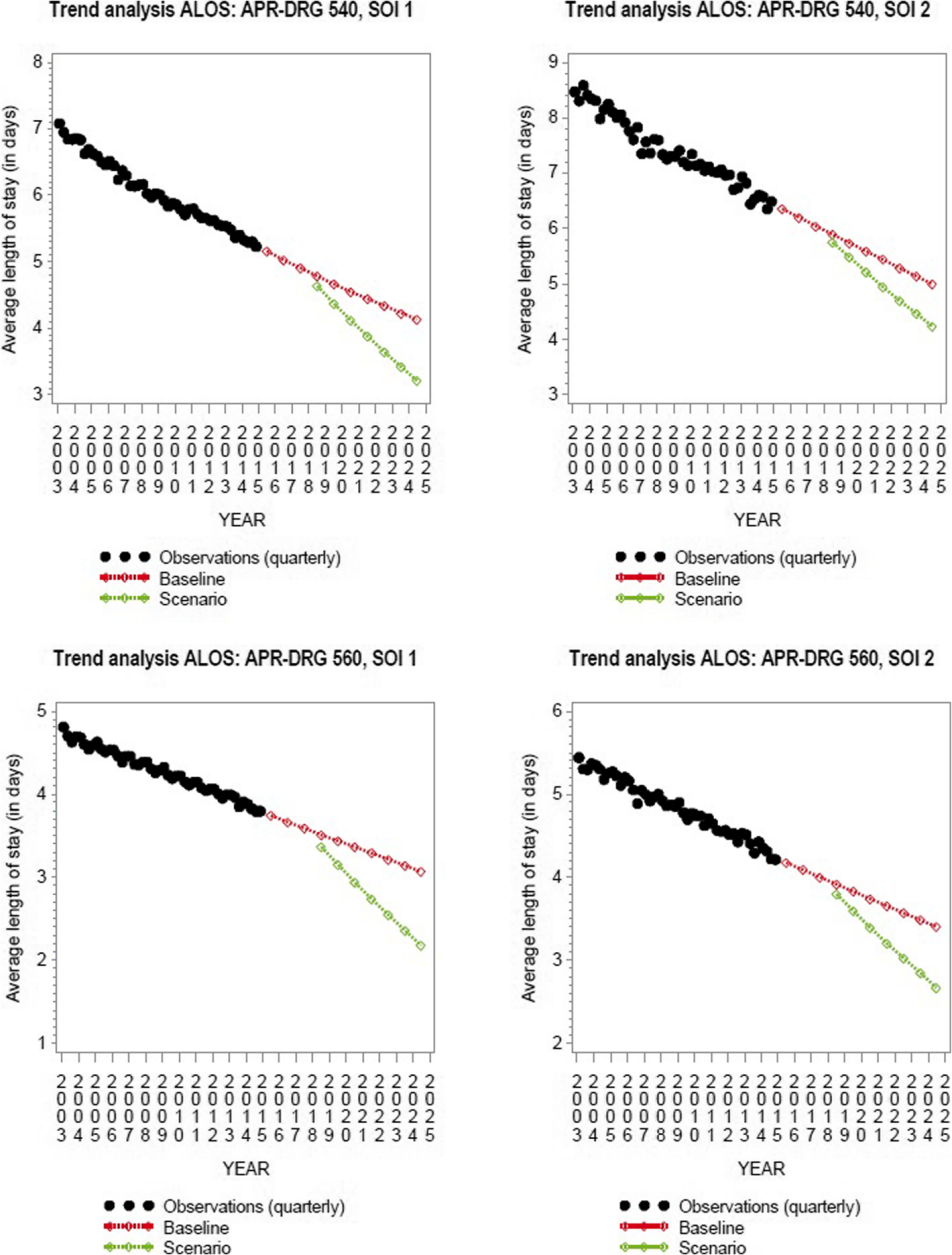
ALOS trend analysis for C-section deliveries (APR-DRG 540) and vaginal deliveries without procedure (APR-DRG 560), SOI 1 and 2, baseline and alternative scenario (2003–2025)

Forecasted ALOS using the alternative scenario (accelerated reduction in ALOS) is shown in Fig. 2 for SOI 1 and 2. Results from the projection model, based on this alternative scenario, are presented in Table 2. With international benchmarks as future targets the required reduction in maternity beds amounts to 425 maternity beds (about 16.3% of the observed bed need in 2014), on top of the reduction projected in the baseline model. This amounts to a reduction of 33.7% (from 2545 in 2014 to 1688 in 2025 beds) in the required bed capacity.

**Table 2:** Alternative scenario forecast results

	2014	2020	2025	Abs. difference 2014–2025	Rel. difference 2014–2025
Scenario results					
Days in maternity beds	650,302	545,279	431,202	− 219,100	−33.7%
APR-DRG 540	158,551	137,861	112,050	−46,501	−29.3%
APR-DRG 560	391,268	315,082	233,706	− 157,562	− 40.3%
Maternity beds	2545	2134	1688	− 857	− 33.7%
Difference baseline – scenario					
Days in maternity beds		−45,781	− 108,756	−108,756	−16.3%
APR-DRG 540		−10,709	−26,629	−26,629	−16.8%
APR-DRG 560		−35,275	− 82,624	−82,624	−21.1%
Maternity beds		− 179	−425	− 425	−16.3%

APR-DRG 540 ‘Caesarean Delivery’, APR-DRG 560 ‘Vaginal Delivery’

## Discussion

Despite an expected increase in the number of deliveries, our projection model indicates that the required capacity for maternity beds in Belgium will decrease by 17% at the horizon 2025. This can exclusively be attributed to the significant expected reduction in ALOS. However, as it was the case in the past, accompanying policy measures (such as financial incentives or the expansion of outpatient postnatal care) are needed to achieve a reduction in ALOS and to realize the projected reduction in bed capacity.

Moreover, our alternative scenario shows that this trend can be accelerated. Indeed, when applying international ALOS for deliveries as benchmark, the required capacity for maternity beds decreases by 33.7%. Additional policy measures could also play a role. For instance, from our analysis we expect the rate of C-sections to increase up to 23.5% in 2025. Targeted policy intervention aiming to limit C-sections to clinically justified situations, following the WHO recommendations, may mitigate this shift [27]. As a consequence, the required capacity for maternity beds could be further decreased if policy interventions (e.g. promoting vaginal birth after a previous C-section, promoting the use of evidence-based clinical guidelines, etc. [28]) target the substitution of C-sections by vaginal delivery.

In addition, we assume an occupancy rate of 70%, because it is the rate applied in the Belgian hospital payment system and used to determine staffing standards. However, this rate is quite low compared to international targets [23, 24]. Raising the occupancy rate would reinforce the decrease in the required capacity for maternity beds.

Our study is innovative because it provides policy makers with detailed and objective projections to address capacity issues, which has not been the case in foreign (e.g. France and England) or past Belgian experiences. To our knowledge, this is the first time such a projection is performed. A study conducted in 2005 in Belgium applied a statistical trend analysis to forecast the required bed capacity ten years later [29]. However, in that study, the change in the number of inpatient stays was entirely related to demographic changes and substitution between inpatient and day-care settings. We performed a time trend analysis on the admission rates to better represent this evolution. Secondly, in the 2005 study, the definition of sociodemographic groups was only based on age, implicitly assuming that only the size of the age group affects the future volume of hospital services. Our definition is based on age, sex, and region of residence. Thirdly, disruptive changes were not accounted for in the 2005 report, whereas our alternative scenario evaluates the potential effect of an accelerated reduction of the ALOS for deliveries, in line with international benchmarks.

The same type of analysis could be informative for other countries or other healthcare domains. Indeed, capacity planning projections provide an early warning of pressure points: what region, pathology, bed type, or medical equipment, is most likely to face an increase/decrease in utilisation that may generate imbalances between supply and demand [12]. Of course, to model detailed hospital capacity need, detailed information on past hospital use for a sufficiently long period is necessary.

It is clear from the results that a drastic capacity reduction of maternity beds is indicated in Belgium. Such a reform brings several points of concern, which also hold for many other countries.

First, as Belgium already has a large number of small maternity services compared to neighbouring countries, it is patent that not only the number of beds should be reduced but also the number of maternity services. However, a balance should be found between economies of scale, accessibility (travel time) and quality. Costs tend to be lower when the size, measured as the number of deliveries, is larger [30–32]. Scientific research on the impact of travel time on adverse outcomes is inconclusive [33], but when accessibility is reduced, part of the health care cost (notably transport cost) is shifted to patients. In addition, this increased cost is not uniformly distributed across the population. Regarding the link between volume and outcome, no clear conclusion can be drawn from scientific literature, especially for low-risk births: some studies [34–36] found lower outcomes for deliveries in small units while others [37, 38] found no relationship between the size of the maternity service and the outcomes. The reform will therefore require a balance between all these criteria.

Second, reduced length of hospital stay shifts the setting of the immediate postpartum recovery from hospital to the home, requiring more outpatient care such as midwives’ visit at home, etc. A further reduction of ALOS in maternity services cannot be safely achieved without investing in outpatient postpartum care. Assuming a reduction of the ALOS for vaginal delivery without procedure (APR-DRG 560) to 3 days, a previous study [11] showed that the increased cost of outpatient care (two additional midwife home care visits in the early postnatal period) is more than compensated by the decreased cost of hospital care. This is true even when per diem hospital costs are corrected for workload intensity. In the context of the Action Plan for a reform of the Belgian hospital payment system, the minister of Social Affairs and Public Health supports seven pilot projects, launched in 2016, focusing on deliveries with shortened length of stay [10]. Mid-term evaluation show encouraging results towards an optimization of the organization of care before, during and after the hospital stay: collaboration between care providers is enhanced and patient satisfaction is high. However, some clinicians raised awareness that postnatal care ensured by midwives may finally be more costly due to many subsequent referrals to gynaecologists [39].

Third, a reduction of bed capacity does not imply a linear reduction of costs. One may think that a lower need for nursing days would reduce the staffing requirement and therefore costs. Nevertheless, if staff is simply reduced in proportion to the number of nursing days, workloads will increase, with possible implications for the quality of care as staff work under increased pressure [40]. It has been shown that workload in maternity services is negatively correlated with length of stay [11]. For vaginal deliveries without complications, over half of the minutes of all nursing time consumed over the entire stay are concentrated on the first two days. Eliminating the last, less work-intense, days may increase overall workload intensity.

## Conclusion

Our study suggests that hospital capacity in terms of maternity beds can be considerably reduced in Belgium, in particular due to the continuing reduction of length of postpartum hospitals stays. Our projection model uses historical and current levels of service provision as a starting point and combines three evolutions (population size and composition, average length of stay per pathology group, and admission rate by age and pathology group) to generate forecasts for future capacity requirements.

According to our projection model, and despite an increase in the expected number of deliveries, the required capacity for maternity beds will decrease by 17% at the horizon 2025 if no further policy action is taken. Accounting for the political willingness to reform maternity services in Belgium, we estimate this required capacity for maternity beds will decrease by more than 30% would the Belgian ALOS reach international standards.

## Additional files

Additional file 1: Forecasted evolution of the rate of C-sections. (PDF 53 kb)

Additional file 2: Statistical Appendix. (PDF 669 kb)

## Data Availability

Hospital data (Minimal Hospital Data (MZG – RHM)) are collected by the Federal Public Service (FPS) for Health, Food Chain Safety and Environment. Access can be requested (https://www.health.belgium.be/fr/sante/organisation-des-soins-de-sante/hopitaux/systemes-denregistrement/rhm) but restrictions may apply to the availability of these data.

## Abbreviations

AHRQ: Agency for Healthcare Research and Quality
ALOS: Average length of stay
APR-DRG: All Patient Refined-Diagnosis Related Group
HCPU: Healthcare Cost and Utilization Project
MZG – RHM: Minimal Hospital Data (Minimale Ziekenhuis Gegevens – Résumé Hospitalier Minimum)
SOI: Severity of Illness
WHO: World Health Organization
