# Supplementary figures and images for: Impact of shortened length of stay for delivery on the required bed capacity in maternity services: results from forecast analysis on administrative data
Source: BMC Health Serv Res. 2019 Sep 5;19:637. doi: 10.1186/s12913-019-4500-8 (PMC6729074; doi:10.1186/s12913-019-4500-8)

## Forecasted evolution of the rate of C-sections

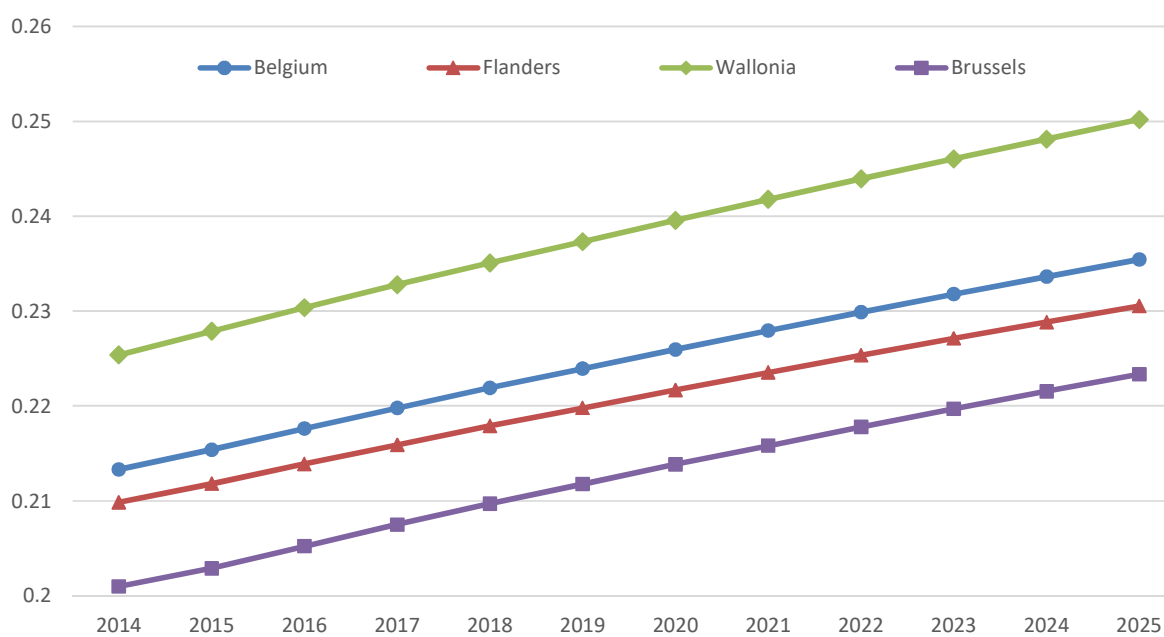

Supplement: Supplementary file 1 — Forecasted evolution of the rate of C-sections. (PDF 53 kb) [file 12913_2019_4500_MOESM1_ESM.pdf]
