# Supplementary material for: Impact of shortened length of stay for delivery on the required bed capacity in maternity services: results from forecast analysis on administrative data
Source: BMC Health Serv Res. 2019 Sep 5;19:637. doi: 10.1186/s12913-019-4500-8 (PMC6729074; doi:10.1186/s12913-019-4500-8)
Supplement: Supplementary file 2 — Statistical Appendix. (PDF 669 kb) [file 12913_2019_4500_MOESM2_ESM.pdf]

This appendix is an excerpt from Van de Voorde et al. (2017).[1]

1 PROJECTION MODEL

1.1 Statistical trend model

Two broad families of statistical models

We estimate the time trend in ALOS and admission rates using specifications from two broad families of statistical models using a different estimation strategy: deterministic trend regression models and auto-regressive integrated moving average (ARIMA) time series models. These two model classes are widely used for planning purposes and satisfy the parsimony principle *'simple models are usually preferable to complex models'* (Diebold, 2007, p. 46).[2] Second, both classes of models have a solid theoretical foundation. Third, they are suitable for generating a large number of forecasts in a semi-automatic fashion within a reasonable computing time. Fourth, the ARIMA models are a general class of time series models which comprehends several popular forecast models as special cases, such as the random walk models or certain exponential smoothing models. A random walk model assumes that every period the forecast value goes randomly up or down by a fixed (estimated) amount. An exponential smoothing model estimates a forecast as a weighted average of past values. The weights are not the same for each time period, but decay over time from the most recent to the most distant time period in an exponential manner. In this way, more recent observations are more important in the computation of the forecast outcome. Exponential smoothing can account for a trend line and seasonality.

Outline of statistical trend analysis

A detailed description of the projection model is given below. An outline is presented in Figure 1. In short, we estimate deterministic trend regression models and ARIMA models on the entire data period. A first selection of models is based on how well a model fits the historical data and its level of complexity. We preserve the best models from both classes. For the final selection, we evaluate the forecast accuracy of all remaining forecast specifications. To this end, we split our data in two periods: an estimation period and a validation period. The former ranges from 2003 to 2011 for inpatient care and from 2006 to 2012 for day care. The validation period goes from 2012 to 2014 for inpatient care and from 2013 to 2014 for day care (see Figure 2).

There are two arguments in favour of this particular split of the data period. First, it is recommended that a validation sample covers at least 20% of the data or about 3 years for inpatient care and 2 years for day care.[3, 4] Second, the estimation period should comprise enough time periods to facilitate an acceptable ARIMA estimation, i.e. at least 30 data points or about 8 years.[5] We re-estimate the models using only data from the estimation period and generate forecast outcomes for the evaluation period. Now it is possible to compare the accuracy of the predictions with actual observations. The models that perform best on this criterion are ultimately chosen. Finally, a combination forecast is computed as an average of the forecasts outcomes from the chosen models, obtained by the estimation on the entire data period.

Objective

The objective of our projection model is to produce the most trustworthy projections of the future. Therefore it has to produce a single forecast model for each dependent variable and forecast group. Moreover, the data used for estimation span a long time period during which health policy, and hospital and healthcare have changed. Hence it is important to account for trend changes in ALOS and admission rates that have set in recent years. Through the use of ARIMA models and a validation period, our methodology gives more weight to recent observations and enduring trend modification in the computation of the forecast outcomes. A good forecast needs to find a balance between the ability of the forecast model to fit the historic data, the accuracy of the forecast outcomes produced by the model and the model's complexity.

The dual selection procedure is specifically set up for this purpose. In the end, it is the accuracy of future forecast that is most important. This is the rationale of the final selection criterion, where forecast accuracy is evaluated using genuine forecasts. Hence the forecast accuracy is determined by assessing how well a model performs on new data that are not used when estimating the model. The caveat is that the validation period is rather short compared to the estimation period as well as the forecast horizon (see Figure 2). It is possible that a specific model performs particularly good or bad in the validation period merely by chance. To this end, we do not select a single best forecast, but rather a group of best forecasts which are averaged out.

Finally, we want to ensure a reasonable level of fit to the historical data, i.e. the ability of the model to reproduce the statistical signal it aims to detect. This is a prerequisite and hence the rationale of the first selection step.

Figure 1 – Outline of the statistical trend model

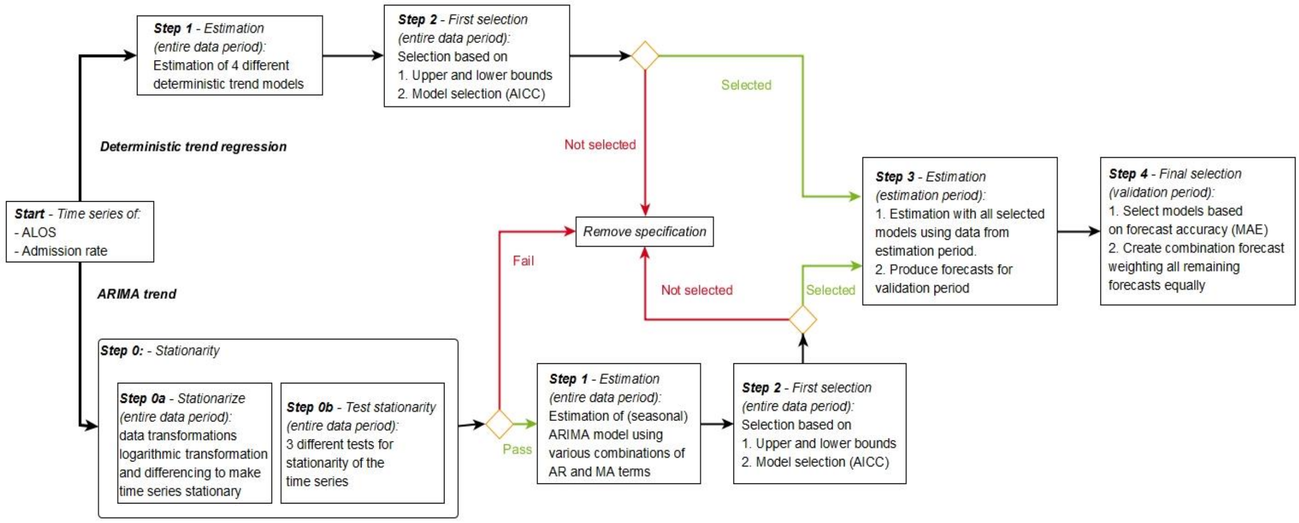

Figure 2 – Overview of time periods

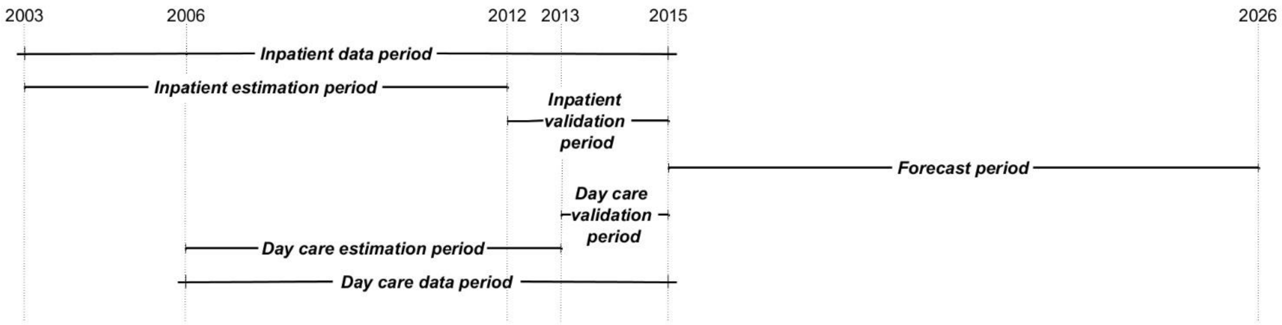

1.1.1 Deterministic trend regressions models

Deterministic trend regressions models assume that there is a time signal to be found in the data. Alterations in ALOS or admission rates are described as a function of time. The time pattern of the forecasted variable has a specific, predetermined functional form, e.g. linear or exponential. The actual fit of the regression line to the data is determined by estimating a finite number of parameters. The main advantage of deterministic trend regression models is that they are clear-cut, easy to estimate and understand. On the other hand, deterministic trend regression models give equal weight to all observations, there is no priority given to the information provided by more recent observations. The latter in combination with the imposed functional form of the time patterns is a stringent assumption that limits the ability of the model to adjust to more complex and dynamic evolutions over time.

Step 1 – Estimation of the time trend

We test four different models, each characterized by two parameters and a specific trend line shape. The first model is a linear trend line. This shape assumes that the absolute change over time remains constant (expressed in days for ALOS or in stays per 100 000 individuals for admission rates).

- A **linear model** of the form: $\boldsymbol{forecast\ variable\ at\ time\ t = A + B \times t}$ where t represents time in quarters, A is a parameter related to the intercept and B is the slope of the linear trend line.

The remaining three models have curved trend lines. They assume that the ALOS or admission rate changes at an increasing or decreasing rate.

- A **logarithmic model**: $\boldsymbol{forecast\ variable\ at\ time\ t = A + B \times \ln(t)}$, where t represents time in quarters, A is a parameter related to the intercept and B is a parameter related to the slope of the natural logarithmic trend line.
- A log-linear model or **exponential model**: $\boldsymbol{ln(forecast\ variable\ at\ time\ t) = A + B \times t}$, where the dependent variable is the natural logarithm of the forecast variable, t represents time in quarters, A is a parameter related to the intercept, B is a parameter related to the slope of the trend line.
- A log-logarithmic model or **power model**: $\boldsymbol{ln(forecast\ variable\ at\ time\ t) = A + B \times \ln(t)}$, where the dependent variable is the natural logarithm of the forecast variable, t represents time in quarters, A is a parameter related to the intercept and B is a parameter related to the slope of the natural logarithmic trend line.

We want to adjust for seasonal patterns if hospital service usage is characterized by seasonality. Hence, for each parametric model, a second version is specified that captures seasonal variation by means of four season dummies, one for each quarter. If, for any reason, a systematic positive or negative deviation from the general long-run evolution occurs in a specific quarter, the season dummy will turn positive or negative, respectively.

The proc reg procedure in SAS is used to estimate the deterministic trend regression models on the time series covering the entire data period. This procedure uses ordinary least squares (OLS) to compute the parameter values. For the exponential and power model, we perform a back transformation after the estimation in order to express the forecast in the original units.

Step 2 – First selection: goodness of fit and acceptable value range

A first selection is made on the estimated models and their forecasts, based on two criteria.

The first criterion imposes an upper and lower bound on the acceptable range of forecast outcomes. Deterministic trend regression models generating forecasts that do not respect the upper and lower bounds are excluded from further consideration. We apply an absolute lower bound of 1 day on the ALOS of inpatient stays (this serves as demarcation between inpatient stays and day care) and of 0 on the admission rates. In addition, we compute a maximum permissible change over time. The upper bound equals the observed maximum value of the time series augmented with the maximum change, whereas the minimum value of the time series minus the maximum permissible change serves as a (second) lower bound. The maximum change is calculated as follows. First, we determine the maximum range of the time series (maximum – minimum). We divide the maximum range by the number of time periods over the entire data period (i.e. 48 quarters for inpatient care, 36 quarters for

day care), to get a maximum equivalent per time period. Next, the maximum equivalent per time period is multiplied with the total number of forecasted time periods (i.e. 44 quarters), resulting in the maximum permissible change during the forecast period. Planning studies in other countries have equally relied on (ad hoc) upper and lower bounds. For example, a hospital capacity planning study in Ontario, Canada, sets projected service volume to zero when the trend projection turns negative.[6] A French planning study employs an upper bound for the number of cataract surgical procedures equal to 80% of the relevant patient population multiplied by two (eyes).[7]

The second criterion carries out a selection based on the fit of the regression model to the historical data. A variety of measures exists to evaluate a model's in-sample goodness of fit. We opt for the Akaike Information Criterion (AIC), corrected for usage in small sample sizes (AICc) (more details can be found in Van de Voorde et al., 2017[1]). The AIC presents a trade-off between the goodness fit of the model and the complexity of the model evaluated by the number of estimated parameters. The AICc adds an additional bias correction in case the number of time periods is rather small compared to the number of estimated parameters. A penalization for the model's complexity is necessary, since a perfect fit can always be obtained by using a model with enough parameters. Such an over-fitted model is not able to distinguish the statistical signal from the random variation and does not produce trustworthy forecasts.

The (corrected) AIC as well as the Bayesian information criterion (BIC) are widely used for assessing ARIMA specifications and regression models with a large number of potential parameters. In the examined deterministic trend regression models, the number of parameters is rather limited, so that the penalization for model complexity might appear unnecessary. However, the AICc can provide guidance whether to proceed with the seasonal version of the deterministic trend regression model or not. As we will use AICc for ARIMA selection, its usage here can be additionally defended for reasons of consistency.

The model with the lowest AICc value offers the best trade-off between goodness of fit and complexity. However, it occurs that the AICc value of an alternative model differs only to a limited extent, suggesting that there is supporting evidence in the data for either model. Benchmarks have been proposed in the literature for assessing whether the difference in AIC between two models is significant. A difference in AICc values of 2 or lower can be considered negligible, whereas a difference of 5 or higher suggests a clear preference for the model with the lower AICc value.[8-10]

Our selection criterion is based on the suggested benchmark value of 5. We compute the AICc value of all seasonal and non-seasonal deterministic trend regression models and identify the minimum value. Regression models whose AICc value differs no more than 5 units with the minimum are considered to fit the data sufficiently well. These models are retained for further analysis.

Step 3 – Estimation on data from estimation period

All deterministic trend regression models that have passed the first selection round are re-estimated on the subset of the data belonging to the estimation period (see Figure 2). Next, forecasts for the validation period are produced by each model. The accuracy of the forecast of each individual model is evaluated in the next step.

Step 4 – Final selection: forecast accuracy

The final selection is made by assessing the forecast performance. We evaluate the accuracy of the forecasts by comparing predictions with the actual observations during the validation period. Predictions from deterministic trend regression models as well as from ARIMA models are assessed in the same procedure. Various accuracy measures exist and may lead to different rankings. We opt for the mean absolute error (MAE), which is a frequently used, scale-dependent measure for forecasting accuracy. Scale dependent means that its value is expressed in the same units as the time series, i.e. days for ALOS and admissions per 100 000 individuals for admission rates. Since we evaluate the accuracy for each time series separately, scale dependence is not a problem. Scale-dependent measures are preferred to scale-independent measures when observations can take values close to zero, which is the case for the admission rates of specific pathology groups. Furthermore, we prefer the MAE to the root mean squared error (RMSE), since it is less sensitive to an occasional large error that might result from random variation. The MAE is computed as the average of the sum of all errors for a given time series without regard to sign (see Eq. (1)).

$$\textbf{Mean absolute error} = \frac{1}{n}\sum_{t=1}^{n}|Y_t - F_t| \quad (1)$$

where t is a subscript representing the n time periods in the validation period, Y_t are the real observations, F_t are the forecasts.

Next, we calculate the mean and standard deviation of the MAE over all remaining models. All models that have a MAE below the mean value augmented with 1 standard deviation are selected as 'accurate' models. We create one combination forecast as the average of the predictions of all remaining ARIMA and deterministic trend regression model. The final forecast is of course based on the estimates using the entire data period.

It has been shown that forecasting accuracy can be improved by combining various forecasts. [11-13] The idea is that several models can perform reasonably well and that each of them has its own strengths and limitations. The resulting combination forecast softens the more pronounced predictions from any one individual model. Similarities can be drawn with the way opinions of different experts/stakeholders are taken into account. In general, one does not favour one particular expert/stakeholder opinion, but attempts to incorporate all provided relevant information. In addition, combining forecasts can be seen as an approach to account for specification uncertainty.

From the combination forecasts, we derive change rates over time which are used to adjust the starting values observed in 2014 to the projected trends.

1.1.2 ARIMA models

The ARIMA class of models assumes that current values of ALOS or admission rates are a function of past events. Two events are considered important here, former values of the forecasted variable and shocks, i.e. deviations from what the ARIMA model considers a 'normal' value, which occurred in the past. In this way ARIMA models can adapt in a more flexible way to the data, and can give e.g. more weight to recent observations.

ARIMA is short for auto-regressive integrated moving average. ARIMA models do not structure the data to fit a pre-determined functional form, they combine three techniques to approximate in a parsimonious and accurate way the complexity of the time patterns in the data. We briefly introduce the three techniques and discuss them more in detail below. The first technique, differencing, is intended to make the data approximately stationary. A time series is stationary if its statistical properties (e.g. mean, variance, autocovariances, etc.) are time-independent. It exhibits no trend, no systematic growth or decline, nor seasonal patterns. Non-stationary data can be stationarized through differencing, i.e. looking at changes over time rather than the actual levels. A time series that requires differencing is an integrated series (the 'I' in ARIMA). The second and third technique are essential in the ARIMA estimation. The value of the (potentially differenced) forecast variable is considered a function of its own lagged values, called the auto-regressive terms (the AR in ARIMA), and lagged values of forecast error terms, called the moving average terms (the MA in ARIMA). The forecast errors are the difference between the prediction of the model and the actual observed value. Each ARIMA specification is adapted to the forecasted variable and can be summarized by three numbers as an *ARIMA(p,d,q)* model. It consists of *p* auto-regressive terms, *d* levels of differencing and *q* moving average terms. A (non-technical) ARIMA equation has the following form:

Differenced forecast variable at time =

> ***constant***
>
> ***and/or the weighted sum of the p most recent values of the forecast variable***
>
> ***and/or the weighted sum of the q most recent forecast errors.***

ARIMA models also have a seasonal version, sometimes called SARIMA, short for seasonal ARIMA. They follow the same main principles as ARIMA models. First, the time series is purged from seasonal patterns to make it stationary. In our setting a season is defined as a quarter. Seasonal differencing does not occur between adjacent periods, but between the same time periods in adjacent years, e.g. the difference is taken between quarter 1 in year 2004 and quarter 1 in year 2003. In the same vein, auto-regressive and moving average terms are included from lagged seasons. SARIMA models are summarized by 6 numbers in the form *(p,d,q)x(P,D,Q)*, with *(p,d,q)* the normal ARIMA terms and *(P,D,Q)* the seasonal counterpart.

The advantage of the ARIMA model class is its ability to produce forecasts in a very flexible and data-driven way that account for time-varying trends and seasonal patterns requiring a limited number of parameters. Many different specifications can be tested and even combined. The disadvantage is that the model parameters are more difficult to interpret compared to parametric regressions.

Figure 1 provides an overview of the followed procedure.

Step 0 – Stationarity of the data

Non-stationary data should be transformed and made stationary. In principle, any nonlinear transformation might be suitable to stationarize a time series. Given the large number of potential transformations, we chose to focus on the two most frequently used techniques, i.e. taking the natural logarithm of the series and differencing. We apply the commonly used rule of thumb of transforming no more than two times in total.[3] Hence, for each time series, we specify eight transformation options:

1. No differencing (d=0; D=0). The time series is not transformed.
2. First-difference transformation (d=1; D=0). A new time series is constructed that consists of the period-to-period changes in the forecast variable. The equation looks like this: ***first-differenced forecast variable at time t = forecast variable at time t – forecast variable at time t-1.***
3. Second-difference transformation (d=2; D=0). A second transformation may be necessary when the first-differenced time series is still non-stationary, e.g. for smoothly-varying time series. In this case the newly defined series takes the first-difference of the first-difference. We only use a second-difference transformation if the first-difference transformation is not stationary. Remark that this is not the same as the change relative to the two period lagged value. The equation looks like this: ***second-differenced forecast variable at time t = first-differenced forecast variable at time t – first-differenced forecast variable at time t-1.***
4. Seasonal first-difference transformation (d=0; D=1). First-differences are taken between two successive seasons aiming to filter out the seasonal pattern. As we work with quarterly data, the new series consists of the change relative to the four period lagged value. The equation looks like this: ***seasonal first-differenced forecast variable at time t = forecast variable at time t – forecast variable at time t-4.***
5. First-difference combined with seasonal first-difference transformation (d=1; D=1). The normal first-difference transformation is combined with a seasonal first-difference transformation. The equation looks like this: ***seasonal and normal first-differenced forecast variable at time t = first-differenced forecast variable at time t – first-differenced forecast variable at time t-4.***
6. Logarithmic transformation of the dependant variable, no differencing (d=0; D=0). This is identical to option 1 but with a log-transformed forecast variable.
7. Logarithmic transformation of the dependant variable, in combination with first-difference transformation (d=1; D=0). This is identical to option 2 but with a log-transformed forecast variable.
8. Logarithmic transformation of the dependant variable in combination with seasonal first-difference transformation (d=0; D=1). This is identical to option 4 but with a log-transformed forecast variable.

Seasonal differencing, i.e. options 4, 5 and 8, consumes at least one entire year of data simply to calculate the differences. These observations are no longer available for the estimation. Given the shorter data period for day care (see Figure 2), we do not allow for seasonal differencing when estimating the trend for admission rates for day care.

The stationarity of the resulting data over the entire data period is controlled for each of the eight specifications. We apply three different tests for stationarity: the augmented Dickey-Fuller test (ADF), the Phillips-Perron test (PP) and the Kwiatkowski-Phillips-Schmidt-Shin test (KPSS).[14, 15] The main reason for using several tests is the low statistical power of each of the tests separately in small samples. Statistical power is the ability of a test to reject a false null hypothesis. The first two tests have the null hypothesis of a non-stationary series, whereas the latter test has the null hypothesis of a stationary series. The opposite null hypotheses are of added value given the possible power issues. The tests are executed in SAS using proc autoreg. For the ADF test, we include the number of lags as proposed by Ng and Perron (2001).[16] The specification for the PP and the KPSS tests are automatically computed in SAS. We assume that the time series is stationary if at least two of the three tests indicate that the series is stationary at the 5% significance level.

All (differenced) time series that are considered stationary are retained for the ARIMA estimation. No forecasts are calculated for non-stationary series.

Step 1 – ARIMA estimation

In the estimation phase, the evolution of the ALOS and admission rates is fitted on the entire data period using combinations of auto-regressive and moving average terms.

A time series is characterized by autoregressive behaviour if it has the tendency to return to its mean. The sum of the coefficients of the auto-regressive terms indicates the speed of mean reversion. The closer the sum is to zero, the faster the series moves back towards its mean; if the sum is close to one, the mean reversion is slow. In the latter case, one should worry about the stationarity of the time series.

A time series exhibits moving-average behaviour if it is influenced by apparent random variability which effects last two or more successive periods. The coefficient of a moving average term related to a lagged period, expresses to what extent the forecast error of that lagged period affects the current period's value. If the sum of moving average terms approaches one, the time series might be overdifferenced.

We comply with two rules of thumb in (business) economics to limit the sum of auto-regressive and moving average terms to three, i.e. $p+q\leq3$, and to limit the number of seasonal auto-regressive and moving average terms to 1, i.e. $P+Q\leq1$.[3] In addition, we limit p and q to 2. More specifically, we estimate the following non-seasonal models where * denotes the degree of differencing in one of the eight transformation options specified above: (0,*,0); (1,*,0); (0,*,1); (1,*,1); (2,*,0); (0,*,2); (2,*,1); (1,*,2). All models are estimated with constant. The SARIMA models that we consider are all non-seasonal models complemented with either x(0,1,0) or x(0,*,1) or x(1,*,0) as seasonal component.

The estimation is executed in SAS using proc ARIMA.[5] The parameters are identified using the maximum likelihood method with a maximum of 200 iterations.

Step 2 – First selection: goodness of fit and acceptable value range

The selection criteria are similar to those used for deterministic trend regression models, and are described in detail in step 2 in section 1.1.1.1. The first criterion imposes an upper and lower bound on the prediction results. The second criterion assesses the goodness of fit, corrected for the complexity of the model using the AICc value. The evaluation is performed separately for each of the eight differencing options. The reason is that differencing reduces the number of data observations in the time series. One major advantage of AICc is its ability to compare very different (non-nested) estimation models, however, with one important premise, being that the underlying data are the same.

Step 3 – Estimation on data from estimation period

Step 3 is completely similar to step 3 for deterministic trend regression models. First, the selected models are re-estimated using only data from the estimation period. For each model, forecasts are produced for the validation period.

Step 4 – Final selection: forecast accuracy

The final selection is made by assessing the forecast performance of all remaining deterministic trend regression models, and ARIMA models. The evaluation of forecast accuracy and final selection is described in detail in step 4 for deterministic trend regression models.

1.2 Projection outcomes

1.2.1 Starting values

Individuals who belong to different socio-demographic subgroups differ with respect to the type of hospital services they use. We account for this by computing the ALOS and the hospital admissions in 2014 – the most recent available year – at the intersection of socio-demographic and pathology groups. This cross-section is the point of departure. The effects of three evolutions – i.e. the demographic evolution, the trend in admission rates, and the trend in ALOS – are applied to the starting values to generate the projection results. For all three variables, the projected future evolution is converted into growth rates over time to easily modify the starting values in 2014. The baseline

forecast results combine the effects of all three evolutions. However, the projection model can equally generate results using only one or two factors and keeping the other factor(s) fixed at the 2014 level.

1.2.2 Projected hospital stays, days and beds

Demographic evolution

The projections for hospital usage are adjusted for the evolution in population size of a socio-demographic group. This is called static reweighting. More specifically, the future number of stays for a certain pathology P and demographic group D in a specific year Y and quarter Q corresponds to the number of hospital stays observed in 2014 multiplied by the growth in population size for group D between 2014 and that year Y in the future.

Equation (2) indicates this further in mathematical terms. It can be shown that static reweighting can be equally expressed in terms of the admission rate of 2014. This is important when the effects of static reweighting are combined with the results of the statistical trend analysis in the following section.

$$\begin{aligned}
&\textbf{total number of stays}_{year\ Y,\ quarter\ Q,\ group\ D,\ pathology\ P} \qquad (2)\\
&= \frac{\text{size}_{year\ Y,\ group\ D}}{\text{size}_{year\ 2014,\ group\ D}} \times \text{total number of stays}_{year\ 2014,\ quarter\ Q,\ group\ D,\ pathology\ P}\\
&= \frac{\text{size}_{year\ Y,\ group\ D}}{100\ 000} \times 100\ 000\\
&\times \frac{\text{total number of stays}_{year\ 2014,\ quarter\ Q,\ group\ D,\ pathology\ P}}{\text{size}_{year\ 2014,\ group\ D}}\\
&= \frac{\text{size}_{year\ Y,\ group\ D}}{100\ 000}\\
&\times \text{admission rate per }100\ 000_{year\ 2014,\ quarter\ Q,\ group\ D,\ pathology\ P}
\end{aligned}$$

Evolution in admission rates

The projected volume in hospital stays is not only affected by demographic evolutions, but also the change in admission rates over time. To incorporate the latter effect, we further adjust the (demographically) reweighted number of stays for year Y, quarter Q, socio-demographic group D and pathology group P by the change rate in admissions as projected by the projection model. The change rate is pathology- and age-specific and is computed as the ratio of the forecasts of the admission rate in year Y, quarter Q to the forecast of the admission rate in 2014, quarter Q. The age groups used for the trend estimation of admission rates are linked to the age definition applied for socio-demographic groups and are denoted D'. The projected total volume in hospital stays for a specific year Y corresponds to the sum over all quarters Q, socio-demographic groups D and pathology groups P. We calculate this separately for inpatient hospital stays and day care. Equation (3) summarizes the calculations.

$$\begin{aligned}
&\textbf{baseline total stays}_{year\ Y} \qquad (3)\\
&= \sum_{Q}\sum_{P}\sum_{D} \frac{\text{size}_{year\ Y,\ group\ D}}{100\ 000}\\
&\times \text{admission rate per }100\ 000_{\ year\ 2014,\ quarter\ Q,\ group\ D,\ pathology\ P}\\
&\times \frac{\text{forecast admission rate per }100\ 000_{year\ Y,\ quarter\ Q,\ pathology\ P,\ group\ D'}}{\text{forecast admission rate per }100\ 000_{\ year\ 2014,\ quarter\ Q,\ pathology\ P,\ group\ D'}}
\end{aligned}$$

Evolution in ALOS

The second outcome of interest is the number of nursing days for each future year Y up to 2025. This is only computed for inpatient stays. The number of nursing days is equal to the product of the baseline total stays in year Y, broken down by quarter Q and pathology group P, and the ALOS observed in 2014 adjusted by the change rate between year Y and 2014. The methodology to include the trend in ALOS is similar to the way the trend in admission rates is incorporated in the model. In order to obtain the yearly number of projected nursing days, a sum is taken over all pathology groups and quarters. This is shown in Equation (4).

$$\begin{aligned}&\textbf{baseline total nursing days}_{year\ Y} \\ &= \sum_Q \sum_P \text{baseline total stays}_{year\ Y,\ quarter\ Q,\ pathology\ P} \\ &\times ALOS_{year\ 2014,\ quarter\ Q,\ pathology\ P} \times \frac{\text{forecast ALOS}_{year\ Y,\ quarter\ Q,\ pathology\ P}}{\text{forecast ALOS}_{year\ 2014,\ quarter\ Q,\ pathology\ P}}\end{aligned} \tag{4}$$

The future number of beds for inpatient stays is the third main outcome variable. They are calculated by bed type (B). Therefore, the admission rate and the ALOS of an inpatient stay is further broken down by bed type. We compute the projected number of nursing days per bed type (Eq. (5)) and apply a bed type specific normative occupancy rate to infer the normative bed need (Eq. (6)). The occupancy rates are detailed in Van de Voorde et al. (2017).[1] For maternity beds an occupancy rate of 70% is used.

The number of day-care places is calculated in a similar way, but without accounting for changes in ALOS. Each day-care stay is attributed a duration of 1 day. In addition, the number of workdays in a day care centre is about 250 and not 365 as in an inpatient facility. For further details on the computation, see Van de Voorde et al. (2017).[1]

$$\begin{aligned}&\textbf{baseline total nursing days}_{year\ Y,\ bed\ type\ B} \\ &= \sum_Q \sum_P \sum_D \frac{\text{size}_{year\ Y,\ group\ D}}{100\ 000} \\ &\times \text{admission rate per 100 000}_{year\ 2014,\ quarter\ Q,\ group\ D,\ pathology\ P,\ bed\ type\ B} \\ &\times \frac{\text{forecast admission rate per 100 000}_{year\ Y,\ quarter\ Q,\ pathology\ P,\ group\ D'}}{\text{forecast admission rate per 100 000}_{year\ 2014,\ quarter\ Q,\ pathology\ P,\ group\ D'}} \\ &\times ALOS_{year\ 2014,\ quarter\ Q,\ pathology\ P,\ bed\ type\ B} \\ &\times \frac{\text{forecast ALOS}_{year\ Y,\ quarter\ Q,\ pathology\ P}}{\text{forecast ALOS}_{year\ 2014,\ quarter\ Q,\ pathology\ P}}\end{aligned} \tag{5}$$

$$\begin{aligned}&\textbf{baseline total required beds}_{year\ Y,\ bed\ type\ B} \\ &= \frac{\text{baseline total nursing days}_{year\ Y,\ bed\ type\ B}}{365 \times \text{occupancy rate}_{bed\ type\ B}}\end{aligned} \tag{6}$$

1.3 References

1. Van de Voorde, C., et al., *Required hospital capacity in 2025 and criteria for rationalisation of complex cancer surgery, radiotherapy and maternity services*, in *KCE Reports*. 2017, Belgian Health Care Knowledge Centre (KCE): Brussels.
2. Diebold, F.X., *Elements of forecasting*. 4th edition ed. 2007, Mason: South-Western Publ.
3. Nau, R., *Statistical forecasting: notes on regression and time series analysis*. 2014.

4. Hyndman, R.J., *Measuring forecast accuracy*. 2014.

5. SAS Institute Inc., *The ARIMA procedure*, in *SAS/ETS 9.2 User's Guide*. 2008, SAS Institute Inc.: Cary, NC.

6. Hay Group Health Care Consulting, *Developing a Health Care Capacity Plan for Ontario – "Proof of Concept"*. 2015, The Ontario Hospital Association: Toronto.

7. Mouquet, M.-C. and P. Oberlin, *Impact du vieillissement sur les structures de soins à l'horizon 2010, 2020 et 2030*, in *Dossiers Solidarité et Santé*. 2008, Direction de la recherche, des études, de l'évaluation et des statistiques (DREES): Paris.

8. Duong, Q.P., *On the choice of the order of autoregressive models: A ranking and selection approach.* Journal of Time Series Analysis, 1984. **5**(3): p. 145-157.

9. Brockwell, P.J. and R.A. Davis, *Introduction to Time Series and Forecasting*. 2016, Cham: Springer International Publishing.

10. Dziak, J., R. Li, and L. Collins, *Critical Review and Comparison of Variable Selection Procedures for Linear Regression*. 2007, Penn State University: State College, Pennsylvania. p. 69.

11. Claeskens, G., *Statistical Model Choice.* Annual Review of Statistics and Its Application, 2016. **3**(1): p. 233-256.

12. Makridakis, S., S.C. Wheelwright, and R.J. Hyndman, *Forecasting: methods and applications*. 3rd ed. 2003, Hoboken: Wiley.

13. Kapetanios, G., V. Labhard, and S. Price, *Forecasting Using Bayesian and Information-Theoretic Model Averaging: An Application to U.K. Inflation.* Journal of Business & Economic Statistics, 2008. **26**(1): p. 33-41.

14. Mahadeva, L. and P. Robinson, *Unit Root Testing to Help Model Building*. Handbooks in Central Banking, ed. A. Blake and G. Hammond. 2004, London: Centre for Central Banking Studies, Bank of England. 53.

15. McCarthy, D. *An Introduction to Testing for Unit Roots Using SAS: The Case of U.S. National Health Expenditures*. in *SAS Global Forum*. 2015. Dallas, TX.

16. Ng, S. and P. Perron, *LAG Length Selection and the Construction of Unit Root Tests with Good Size and Power.* Econometrica, 2001. **69**(6): p. 1519-1554.
